# Impact of the dispersive patch placement on dissipated power in radiofrequency ablation for pulmonary vein isolation via a virtual patient study

**DOI:** 10.1038/s41598-025-90158-1

**Published:** 2025-02-27

**Authors:** Minha Anees, Zoraida Moreno Weidmann, David Viladés Medel, Jose M. Guerra, Luca Gerardo-Giorda, Argyrios Petras

**Affiliations:** 1 Johann Radon Institute for Computational and Applied Mathematics (RICAM), Austrian Academy of Sciences, Linz, Austria; 2 Department of Cardiology, Hospital de la Santa Creu i Sant Pau, IIB Sant Pau, Universitat Autònoma de Barcelona, CIBER CV, Barcelona, Spain; 3 Institute for Mathematical Methods in Medicine and Data-Based Modelling, Johannes Kepler University, Linz, Austria

**Keywords:** Biomedical engineering, Translational research, Mathematics and computing

## Abstract

Radiofrequency ablation (RFA) is a minimally invasive technique for treating arrhythmias by interrupting abnormal electrical signals in the heart. Through a catheter tip, it delivers an alternating current that flows through the heart muscle tissue and the blood to a dispersive patch on the patient’s skin. This study aims to test the hypothesis that the placement of the dispersive patch affects the efficacy and safety of RFA. By optimizing the patch position, the procedure could be made more effective and less risky for patients. A 3D in-silico model, based on patient imaging data, was developed to examine the effects of dispersive patch (DP) positioning on electric field distribution within cardiac tissue and the torso during RFA. We conducted 80 computer simulations using a CT-segmented torso model, exploring various DP and electrode configurations while applying standard (25 W) and high (90 W) power settings. For each configuration, we assessed the effectiveness of the DP in delivering power to cardiac tissue near the electrode. The main finding indicates that DP efficacy is significantly influenced by the current delivered to cardiac tissue. Notably, using an anterior patch during ablation proved more effective for the posterior left atrium compared to a posterior patch.

## Introduction

Catheter ablation is a standard treatment for atrial fibrillation (AF). Typical ablation strategies for the termination of AF consist of the isolation of the pulmonary veins (PVI), which can be followed by connecting ablation lines at the roof of the left atrium^1^. This combination typically suffices to effectively eliminate the rapid and irregular electrical signals in the atria, restoring a normal heartbeat. A common approach to perform PVI uses radiofrequency ablation (RFA) with open-irrigated catheters that deliver alternating current in a monopolar way. A dispersive patch (DP) placed on the patient’s skin, typically on the back or thigh, completes the electrical circuit and returns the current from the active electrode.

During the procedure, a catheter is inserted through the patient’s groin, traverses the inferior vena cava (IVC) into the right atrium, and pierces the interatrial septum to enter the left atrial chamber. The catheter is then used to perform point-by-point ablation lines for the isolation of the pulmonary veins.

RFA for PVI can be performed using either standard or high-power protocols. High-power short-duration (HPSD) ablation uses a power typically between 70–90 W for 4–7 s^2^ to eliminate arrhythmogenic tissue. This method leads to faster and more efficient procedures, creating lesions quickly and effectively, thereby reducing procedural time. HPSD ablation has the potential to reduce collateral tissue injury^3^. In contrast, standard power ablation typically uses 25–35 W for 20–60 s^4^.

The RFA process involves two distinct heat generation mechanisms: resistive and conductive heating. Resistive heating occurs in the tissue at a close proximity to the electrode, which propagates deeper in the tissue during the conductive phase. Standard power ablation strategies primarily rely on conductive heating to create lesions, while resistive heating is the main driver for lesion generation in HPSD protocols^5^.

Irreversible tissue thermal damage during the procedure is caused by the heat generated by the electric power delivery^6^: the power dissipated within the tissue is the heat source that induces temperature rise therein^7^. Hence, variations in the power dissipation are a good proxy for variations in the temperature. Energy delivery plays a crucial role in the efficacy and safety of the treatment. If excessive, it may lead to procedural complications, such as tissue overheating and steam pops or the induction of thermal damage on nearby tissues, including the esophagus and the phrenic nerve. On the other hand, inefficient energy delivery may lead to lack of lesion durability and potential redo procedures. To-date, pulmonary vein reconnection of at least one vein after the first radiofrequency ablation procedure occurs in about 56% of patients^8^, with some veins more prone to reconnection than others^9,10^, without any consensus on the reason why such reconnection is observed. Hence, the identification of the impacting factors on the power dissipation in the tissue is essential for the design of ablation protocols, in order to devise an effective and safe treatment plan.

The DP location is an important yet underappreciated factor that affects the efficiency and safety in RFA, and can significantly alter the electric current path through the patient’s torso^11^. On the one hand, it may direct stronger RF current through nearby tissues, such as the esophagus, producing unwanted heating, collateral thermal damage and potential complications^11^. On the other hand, when located opposite to the catheter tip, it can enhance the effectiveness of the procedure by influencing the local current density near the ablation site, resulting in deeper and larger lesions^12^.

A previous clinical study on 20 patients found no notable changes in some ablation markers (total impedance, voltage, current flow and tip temperature), when comparing interscapular and left thigh dispersive patch locations^13^. While these are important markers, this clinical study didn’t report any procedural complications or any information on potential redo procedures on these 20 patients. Another experimental study on in-vivo sheep explored the impact of dispersive patch placement on lesion size^12^, showing that optimal patch placement produces deeper and larger lesions. However, results from animal models cannot be directly applied to humans, since the torso anatomy can play a crucial role in monopolar RFA. To this end, an in-silico study attempted to explore the electric field distribution in the tissue^14^, however using an oversimplified and anatomically incorrect model geometry featuring a spherical heart.

In the present work, we developed a highly detailed anatomically accurate 3D in-silico model based on thoracic patient CT imaging data, which includes the four-chamber heart and the surrounding organs within the torso. We investigate the impact of various dispersive patch positions on ablation markers, such as baseline impedance and power dissipation within the tissue and torso, for typical placements of the catheter in the left atrium during PVI. Using such a detailed model, we aim to provide a deep understanding on how different parameters influence the efficacy and safety of RFA procedures.

## Methods

### Model geometry

**Fig. 1:**
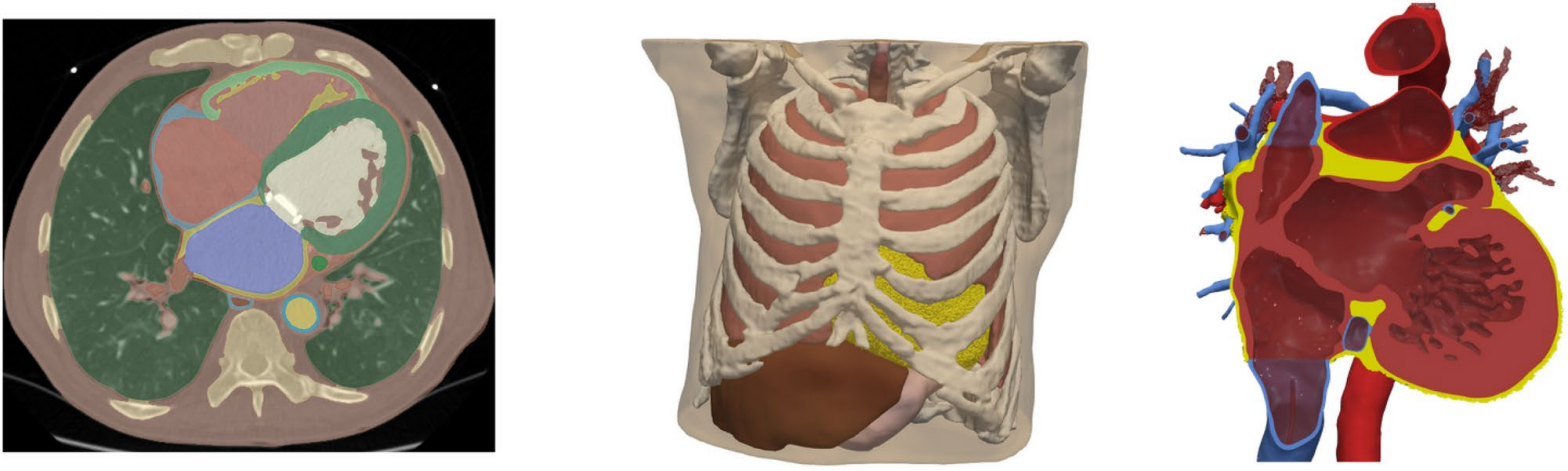
Left: The CT scan image with our multi-label segmentation, consisting of a total of 35 distinct labels identifying each anatomical structure. Middle: The discretized torso geometry with all organs, using tetrahedral elements. Right: The detailed cardiac geometry with trabeculae, pericardium, blood vessels and their walls.

Our 3D computational model is based on a 73 years old male patient imaging data ( BMI—body mass index : 23.5 kg/m^2^, BSA—body surface area : 1.8 m^2^) from the Hospital de la Santa Creu and Sant Pau in Barcelona (Spain), obtained with a Philips Brilliance iCT 256 CT scanner. We performed a detailed segmentation which consists of 35 domains, including spleen, kidneys, bones, lungs, liver, stomach, blood vessels, esophagus and heart among others, as shown in Fig. 1. In particular, our segmentation captures the complex anatomical structural details of the heart, consisting of the four-chambers of heart, trabeculation, blood pools, as well as blood vessels and their walls.

For regions where the pericardium is not visible (near the right atrium), we use the mean thickness reported in the literature. Specifically, in^15^ its shape is compared to a flask-shaped bag with an approximate thickness of 1.5 mm. Our segmentation includes both the interior space (lumen) and outer structure (wall) of the esophagus following available data in the literature^16^.

For blood vessel wall thickness, including the superior and inferior vena cava, aortic wall, coronary sinus, pulmonary veins, and pulmonary artery, values from the literature were used^17–22^. In cases the atrial wall was not visible in the scan, we used available literature data for the wall thickness^23^. The segmentation was done using 3D Slicer software^24^.

We discretized the multi-labeled segmentation into an unstructured tetrahedral finite element mesh using studio software developed by NumeriCor GmbH (https://numericor.at). Figure 1 shows the multi-labeled segmentation as well as the resulting finite element mesh of the full torso with the internal organs and the anatomical details of the heart considered in our reconstructed geometry.

### Dispersive patch and electrode

**Fig. 2:**
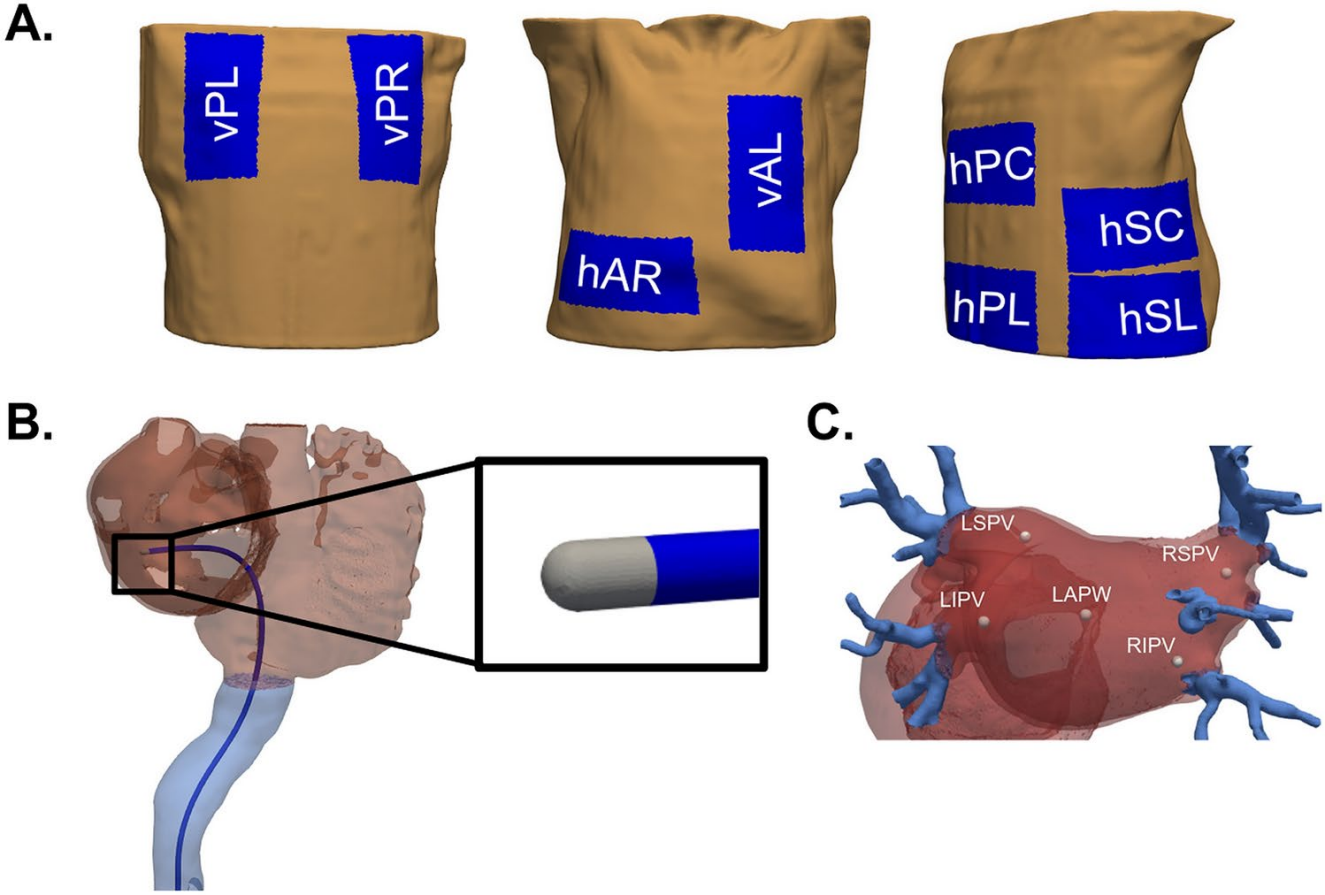
(**A**) Dispersive patches were positioned in eight distinct locations on the torso. Vertical patches were placed on the posterior left (vPL), posterior right (vPR) and anterior left (vAL) positions. Horizontal patches were placed posteriorly across the lower back (hPL) and center back (hPC), on the lower (hSL) and center (hSC) part of the right side as well as on the anterior lower right side (hAR). (**B**) A visualization of our geometry featuring a spherical tip electrode catheter embedded in the right superior pulmonary vein (RSPV). (**C**) The electrode is placed in 5 locations within the left atrium. These are near the right inferior pulmonary vein (RIPV), right superior pulmonary vein (RSPV), left inferior pulmonary vein (LIPV), left superior pulmonary vein (LSPV) and on the left atrial posterior wall (LAPW).

The catheter traverses at the centerline of the inferior vena cava, enters the right atrium (RA), pierces the interatrial septum and is placed on the left atrial wall, following typical catheter placements during PVI. It features a spherical tip electrode of 7 F and 3.5 mm length as in^7^, following designs available in the market. The electrode is placed perpendicular to the left atrial wall at a depth of 0.5 mm within the wall. The catheter and electrode geometries were created using the Salome software (https://www.salome-platform.org), and were embedded within the torso geometry using studio software as shown in Fig. 2B.

We placed a dispersive patch of 8–16.75 cm^2^ in eight different locations on the torso geometry, considering different orientations (horizontal or vertical). The vertical return electrodes were placed on the left (vPL) and right (vPR) sides of the upper posterior, and on the left side of the anterior torso (vAL). The horizontally oriented patch is placed on the lower (hSL) and center (hSC) position of the right side of the torso, as well as on the posterior lower back (hPL), center (hPC) and anterior right (hAR) of the torso. Figure 2A shows the locations of all dispersive patches.

### Mathematical model

A quasi-static electrical potential equation describes the electric field generated during RFA. The equation takes the form

1 $$\begin{aligned} - \nabla \cdot \sigma \nabla \Phi = 0, \end{aligned}$$

where $\Phi$ is the electrical potential and $\sigma$ the electrical conductivity. The equation is solved in the entire geometry, with each subdomain featuring a different electrical conductivity (see Supplementary Material).

A voltage $V_0$ is applied on the upper boundary of the electrode, tuned to achieve the desired power for constant power ablation^7^. The return electrode acts as a ground and closes the electric circuit. Electrical insulation is applied on all the remaining boundaries. More information on the mathematical model appear in the Supplementary Material.

### Simulation protocol

Following commonly used electrode placements during PVI^1,25^, we positioned the electrode on 5 distinct positions of the left atrium: the left atrial wall near the right inferior pulmonary vein (RIPV), right superior pulmonary vein (RSPV), left inferior pulmonary vein (LIPV), left superior pulmonary vein (LSPV) and the left atrial posterior wall (LAPW) (see Fig. 2C). We performed simulations for each position of the electrode (5 electrode positions) using a single patch on a selected location (8 patch locations). Two constant power ablation protocols were considered following typical protocols used during PVI: a standard of 25 W and a high-power of 90 W^26^.

## Results

A total of 80 simulations were conducted for both standard and high power ablation protocols, with each protocol featuring 40 simulations, accounting for the five electrode positions and eight different patch locations. We perform numerical simulations using the finite element method, implemented in FEniCSx (https://fenicsproject.org) open source software.

### Localization of power distribution

**Fig. 3:**
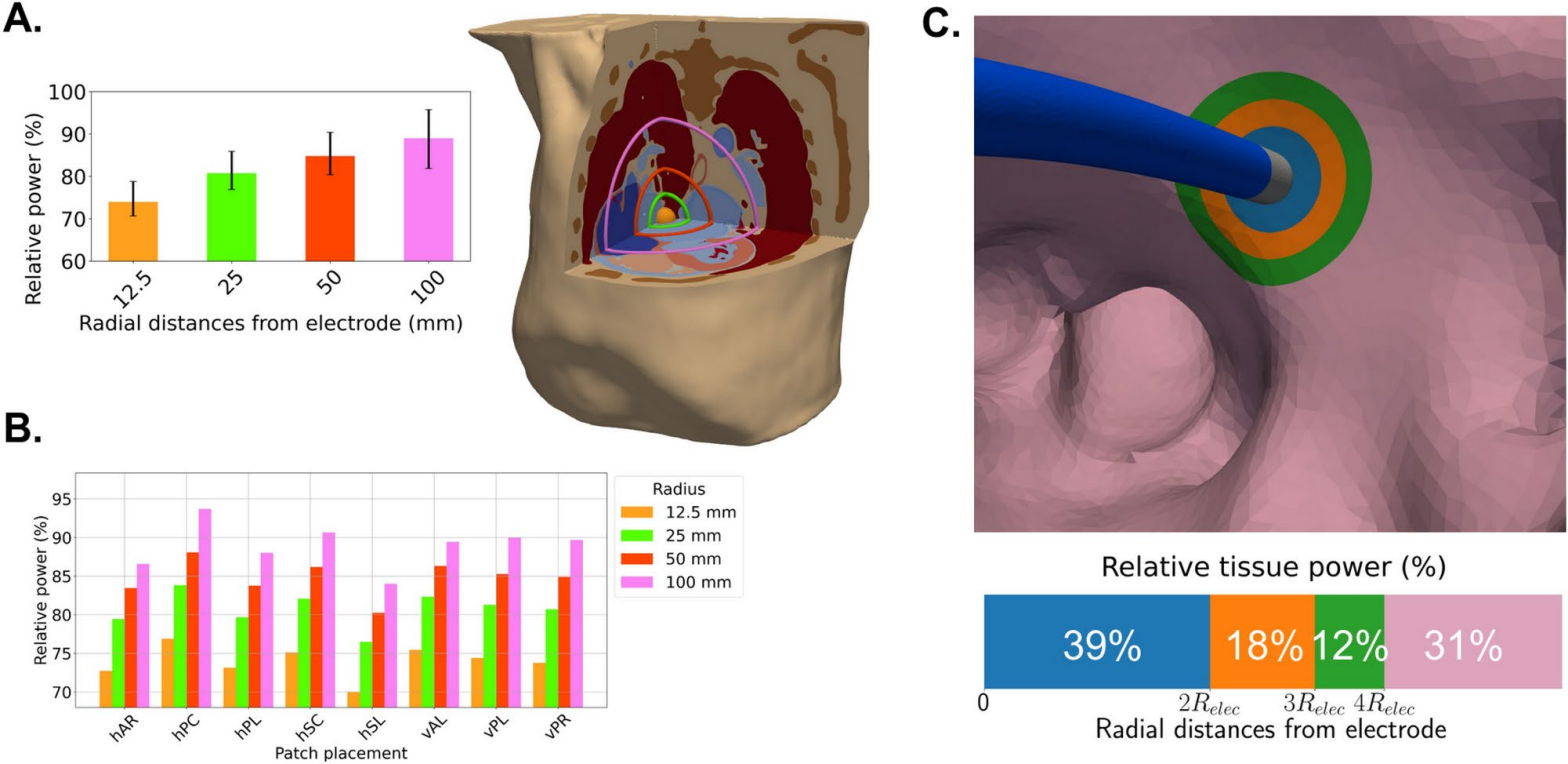
(**A**) Relative dissipation of the total power (%) within different radial distances from the electrode: 12.5 mm, 25 mm, 50 mm, and 100 mm, visualized on the right side of the panel in the case of the RSPV electrode location. As the distance from the electrode increases, more tissue types exhibiting heterogeneous conductivities are encompassed. Bars indicate the average across all ablation protocols, electrode and DP positions; black lines indicate the range of the values. (**B**) Relative dissipation of the total power (%) within the same radial distances from the electrode as in panel A, for the different DP locations. Bars indicate the average across all ablation protocols and electrode positions. (**C**) Relative dissipation of the left atrial tissue power (%) with respect to different radial distances from the electrode, expressed in multiples of the electrode radius $R_{elec}$. The blue region encompasses the atrial tissue within $2R_{elec}$ from the electrode. The orange and green regions encompass the atrial tissue between $2R_{elec}$ and $3R_{elec}$, and between $3R_{elec}$ and $4R_{elec}$ from the electrode, respectively. The pink region consists of the remaining left atrial tissue, as visualized in the upper part of the panel.

At first, we explore the localization of the power dissipated within the entire torso, by considering 4 spheres centered at the electrode location with progressively increasing radius from 12.5 mm to 100 mm. The average relative power dissipation of the total power within each of the spheres for all electrode positions and patch locations is shown in Fig. 3A. At a radius of 12.5 mm, the relative dissipation of the total power inside the torso is approximately 78%, indicating that a significant amount of power is concentrated near the electrode. When this radius is doubled to 25 mm, the relative dissipation only increases by 6% to about 84%. At a radius of 50 mm, the relative dissipation reaches about 88%, reflecting a further increase of only 4% from the 25 mm radius. Finally, at a radius of 100 mm, the relative dissipation reaches approximately 92%. From 50 mm to 100 mm just 4% dissipated as shown in Fig. 3A.

Figure 3B shows the average total power distribution within the torso for each of the 8 different dispersive patch placements considered. The patch placement affects localization of the power dissipation within the torso. Up to a radius of 12.5 mm, an increase of 11% is observed between the lowest and the highest values of the average dissipation of the total power (hSC and hPC patch locations respectively). The least localized power dissipation appears for the horizontal side lower patch, with more than 13.5% of the total power dissipated outside a radial distance of 100mm from the electrode, while the most localized dissipation appears at the horizontal posterior center placement (hPC). Vertical patches display a nearly uniform power distribution within the torso at all considered radii.

The smallest variation with respect to the power dissipation within a radial distance of 12.5 mm from the electrode appears for the hPC position (with values ranging from 76.8 to 77.2% for all electrode positions and ablation protocols), while the largest variability appears for the hSC position (with values ranging from 73.6 to 76.8%). The variability in the relative dissipation of the total power within 12.5 mm, 25 mm, 50 mm and 100 mm radius for all electrode positions is shown in the supplementary material. Negligible differences are observed in the relative dissipation between standard and high power protocols.

Focusing on the relative dissipation of the left atrial tissue power, Fig. 3C shows its distribution at the different colored areas, corresponding to areas within some radial distances from the electrode. In particular, the blue area lies from the electrode up to $2R_{elec}$, the orange one extends from $2R_{elec}$ to $3R_{elec}$, the green from $3R_{elec}$ to $4R_{elec}$ and the pink outside $4R_{elec}$, where $R_{elec}$ is the radius of the electrode. We observe that 39% of the power is dissipated within a distance of twice the electrode radius $2R_{elec}$ in the tissue. More than $50\%$ of the left atrial tissue power is dissipated within a radial distance of up to $3R_{elec}$, which reaches almost $70\%$ for distances up to $4R_{elec}$. The distribution is minimally affected by the dispersive patch placement, with variations of less than 1% as seen in Figure 1 of the supplementary material.

### Cardiac tissue power dissipation

**Fig. 4:**
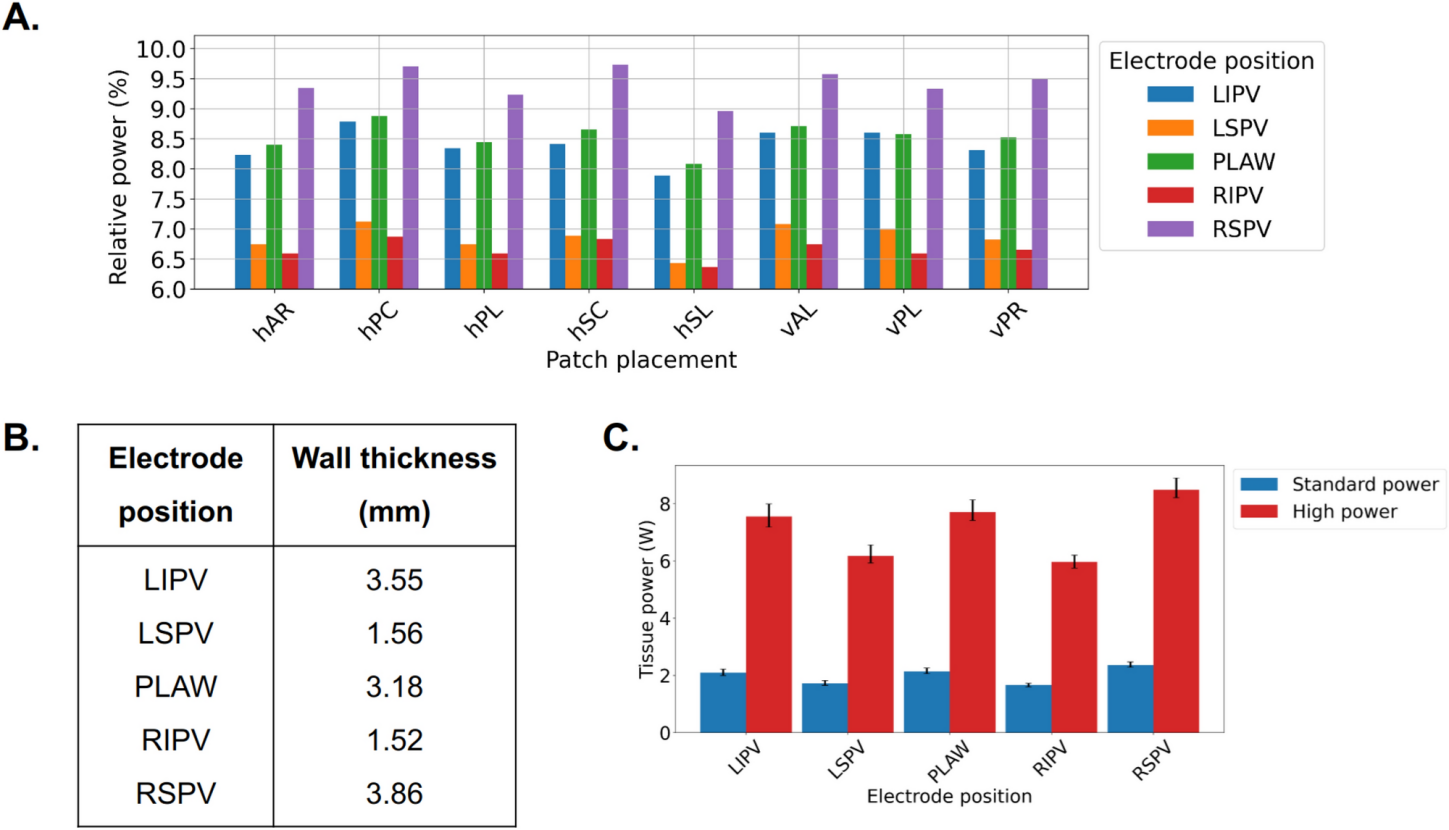
(**A**) Relative dissipation in the left atrium of the total power (%) for all electrode positions and DP placements (standard power protocol). (**B**) The wall thickness for each of the 5 selected electrode locations. (**C**) Power dissipated in the left atrial tissue for standard (in blue) and high power (in red) ablation, for different electrode positions. Bars indicate the average across all DP positions; black lines indicate the range of the values..

Despite having a minimal effect on the localization of the power distribution within the tissue, the dispersive patch location affects the power dissipated in the tissue. As shown in the Fig. 4A, different locations of the patch result in the largest left atrial tissue power dissipation for different electrode placements, with the exception of the hSL location, in which the tissue power is the lowest for all considered electrode positions. The hSL shows approximately 7% to 20% less power dissipated compared to the hPC. Moreover, the power dissipation in the tissue at the RSPV position is the same for both patch location the hPC and hSC.

We also observe a significant decrease in the power dissipation within the cardiac wall for ablations near LSPV and RIPV. By further inspecting the ablation sites, we observe that the wall thickness in these locations is about 1.5 mm, in comparison to the other sites that are thicker than 3 mm (as shown in Fig. 4B). The thickest wall appears at the ablation site near RSPV, which is consistent with the largest power dissipation in the tissue. The tissue power increases by about 41% from the thinner to the thickest ablation site.

**Fig. 5:**
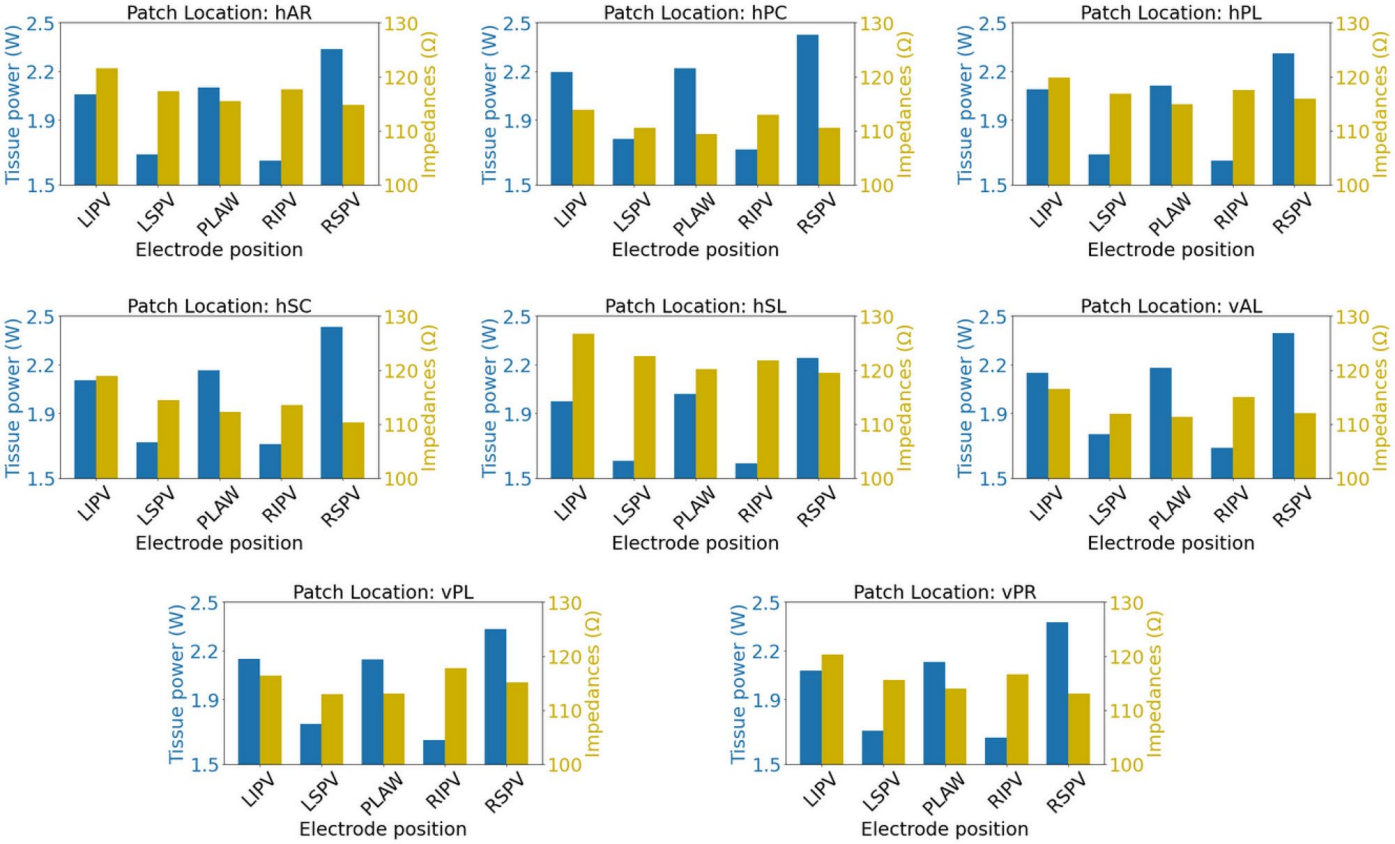
Tissue power and baseline impedance across different electrode positions and patch locations. On the left y-axis in blue color is tissue power, while on the right y-axis in yellow color is baseline impedance.

Furthermore, we explore the possibility of a correlation of the cardiac tissue power to the baseline impedance of the system for different patch locations and electrode positions (shown in Fig. 5). We observe that there is no consistent correlation of the two quantities for a fixed patch location across all different electrode positions. At hPC patch location, the lowest impedance is observed for all electrode positions, while the tissue power dissipation is the highest across all different patch locations.

Similar results were also observed for higher power ablation, as shown in Figure 2 in the Supplementary Material.

### Standard versus high power ablation protocols

High-power ablation protocols significantly increase tissue power dissipation compared to standard power ablation. High-power ablation protocol results in about 250–290% tissue power dissipation when compared to a standard one. This increased power dissipation with high-power ablation allows for more effective and faster lesion formation. This translates to improved efficiency and shorter procedural times.

On the other hand, high power ablation protocols magnify the differences in tissue power observed by other factors, such as the relative electrode—dispersive patch placement. For standard ablation, the difference in power dissipation between RIPV and RSPV is approximately 35%, which is similar to the high power, about 30%, as shown in Fig. 4C. Despite having a comparable percentage of power increase, the absolute power increase is about 0.7 W vs 2.4 W in the standard and high power cases respectively. Due to the typical short duration of the high power ablation protocols, the margin of error becomes much smaller, since small changes in the ablation time may result in very large changes in the power dissipated in the tissue, which may lead to complications.

### Esophageal tissue power dissipation

**Fig. 6:**
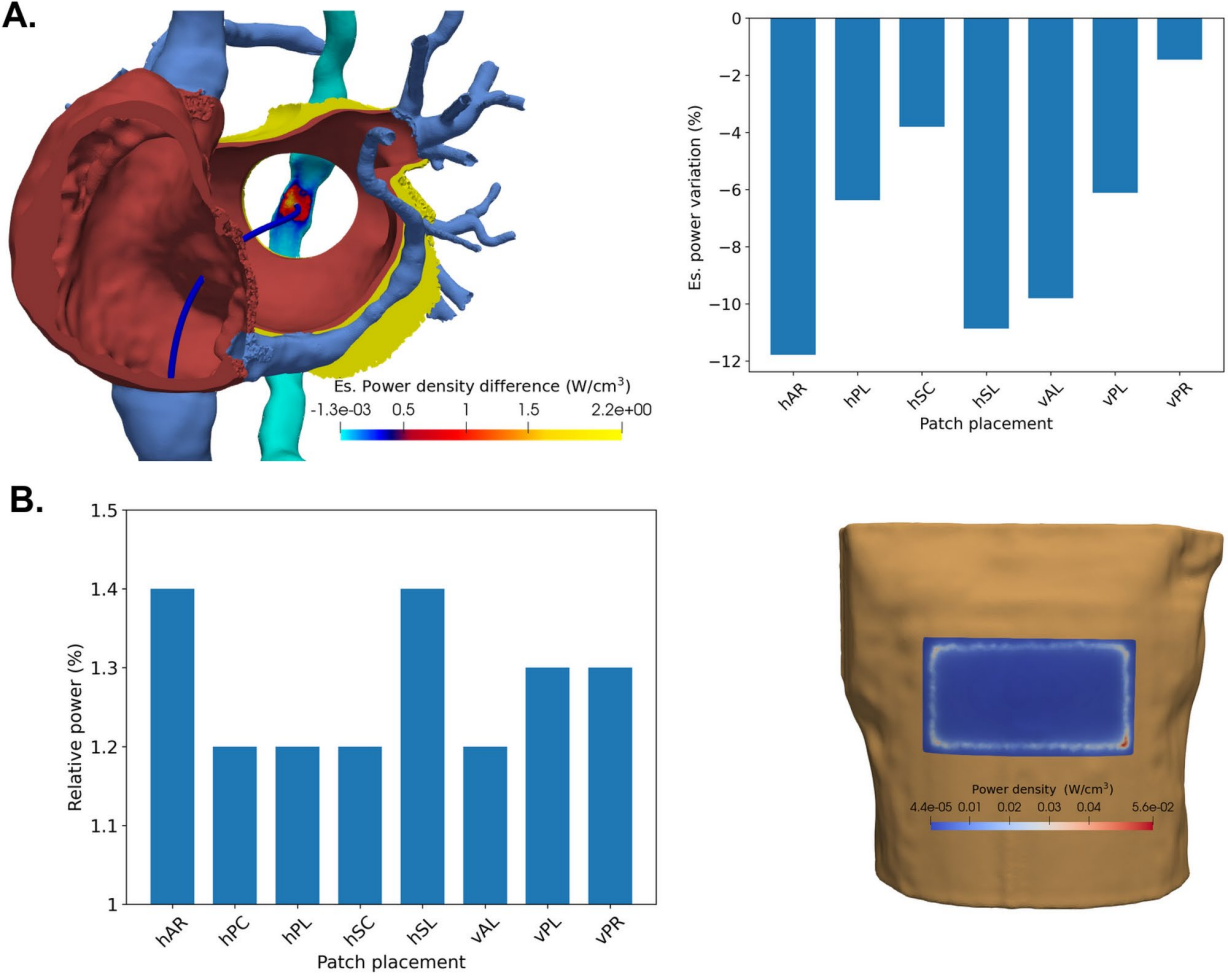
(**A**) Left: Variations in esophageal power density between the vertical anterior left (vAL) and horizontal posterior centre (hPC) patch placement. Positive values indicate larger esophageal power dissipation using the hPC patch. Right: The variation of the percentage of power dissipated inside the esophageal tissue for various patch placements compared to the standard hPC patch location, for an electrode placement near PLAW. (**B**) Left: Relative dissipation of the total power (%) in the vicinity of the patch. For all DP placements, variations are negligible across electrode positions and ablation protocols. Right: The power density distribution at the hPC patch position for the electrode at RIPV..

Different power dissipation patterns based on the patch location are observed for an electrode placement near PLAW. When compared to the commonly used hPC patch position, all the other locations provide a lower power dissipation in the esophageal tissue (see right panel of Fig. 6). Using the hAR, hSL, and vAL patch positions resulted in approximately 11.9%, 10.9%, and 9.8% less power dissipation compared to the hPC respectively.

The cardiac tissue power dissipation using a patch in the vAL position is about the same as in the hPC case (difference less than 2%), however the practical use of this location might be limited in the current practices. On the other hand, the hAR position offers a small reduction in tissue power of about 5%, however it provides a decrease in the esophageal power dissipation of more than 10% Finally, the hSL position reduces the tissue power by about 9%. Similar trends are observed for both standard and high power ablation protocols.

### Power dissipation near DP

We further explore the relative power dissipated in the vicinity of the DP (the area up to a distance of 5 mm from the patch position), for each of the considered positions. As shown in Fig. 6B, the relative percentage of power dissipated near the DP for all patch locations and electrode positions ranges from 1.1 to 1.4% of the total power. Overall, no notable differences were observed in the relative power for different electrode positions or ablation protocols.

The lowest power dissipation is observed at hSC, while the highest power dissipation occurs at hAR, which is 21.6% higher than hSC. Distant patches on the right side of the patient, like hAR and hSL, seem to have a larger power dissipation than other placements. The highest power density areas appear at the border of the DP, and in particular at corner areas.

## Discussion

This study explores the impact of the position of the dispersive patch in the power delivery during PVI via RFA, using a virtual human patient model. Our work presents the first model that accurately captures anatomical features in an unprecedented detail, and simulates an accurate placement of the catheter and dispersive patch, effectively performing a virtual ablation procedure on a digital twin. The power dissipation is an important marker, responsible for the production of heat generation within the tissue, effectively driving the generation of the thermal lesion^7^. Inadequate power delivery can lead to insufficient heating, resulting in incomplete ablation and the potential need for repeat procedures. Maintaining optimal power levels is therefore critical to the efficacy and safety of the treatment.

### Physiological response of virtual model

Our model uses electrical conductivity values from the most comprehensive database for human organs, tissues and fluids^27^, recommended by policy makers, such as the US Food and Drug Administration (FDA). Individual variability would definitely impact the choice of model parameters. Nonetheless, the model successfully predicts a total impedance that lies within the range of the observed baseline values for PVI procedure using both standard and high-power ablation protocols^28,29^.

### Impact on baseline impedance

Our results show that the difference in the baseline system impedance is small for various dispersive patch positions. This is in agreement with the recorded baseline impedance on patients, by placing the return electrode either on the interscapular space or on the thigh^13^.

Another in-vivo experimental study^12^ explored the impact of the dispersive patch location on the resulting lesion size, by placing it either in an optimal location with respect of the electrode or in the opposite side of the thorax. The study found consistently smaller lesions for the non-optimal placement of the patch, with minimal variations in the total impedance of the system. This result comes in agreement with our findings, where we observe that a non-optimal patch location results in a smaller power dissipated in the tissue. A particular example of this trend is the placement of the patch at the lower side of the thorax (hSL), where we consistently obtain smaller power with respect to any other position.

### Efficiency

To improve the efficiency of standard RFA for PVI, a power of 30–35 W was applied for anterior ablations compared to a 25 W for posterior in the SHORT-AF trial^4^ or 50 W anterior and 40 W posterior in another patient study^30^. Our work shows that a potential factor for this discrepancy might be the position of the dispersive patch, which may lead to differences in the tissue power dissipation of up to 11%. Unfortunately, no information regarding the return electrode location is provided in these trials.

Other factors that may affect the efficacy of RFA is the wall thickness. HPSD ablation demonstrates efficacy for ablating thin-walled structures like the atrium and tissues surrounding the pulmonary veins. However, its effectiveness diminishes when applied to thicker tissues, such as those found in the atria around the mitral annulus, a crucial area for mitral valve interventions^26^. For thicker walls, conductive heating allows for deeper lesion creation, thus typical ablation strategies require lower powers than 90 W (for example 40–50 W) for a longer duration, as mentioned in the QDOT-by-LAWT trial^30^. Our results indicate that much lower power is dissipated in the tissue for thin cardiac walls (of about 1.5 mm) in comparison to thicker ones (of approximately 3.5–3.8 mm). High power ablations can mitigate this effect for thin tissues, effectively creating transmural lesions. On the other hand, despite the increase in the tissue power, thermal conduction is necessary to drive the lesion deeper in thicker tissues.

### Safety

The dispersive patch location may alter the power dissipated in the tissue, which can lead to variations of even 12% for HPSD. This variation may result in severe risks of tissue overheating if a constant power ablation protocol is used. Additionally, due to the short duration, the error margin is quite small to avoid tissue overheating. To this end, catheters that deliver HPSD protocols typically include thermocouples, allowing for power reduction as well as increased saline irrigation rates upon high temperature detection^31^.

Various strategies are available aiming to minimize esophageal injury during catheter ablation, such as active esophageal cooling^32^. The very short ablation duration during HPSD protocols appears to be associated with a lower risk of esophageal thermal injury when compared to standard protocols^33^. Yet, esophageal injury may still occur with very high power ablation protocols of 90 W^34^.

Another approach to avoid esophageal damage is the reduction of power dissipation therein. The positioning of the dispersive patch anteriorly for PVI has been proposed in^35^, which redirects RF current away from the esophagus. Our results indicate that indeed such an approach would be beneficial in the reduction of the power dissipated in the esophageal tissue, especially when compared to the standard interscapular positioning or other posterior placements of the return electrode. Particularly, our results show that a horizontal anterior (hAR) positioning of the dispersive patch may reduce the power dissipated in the esophagus with a small decrease in the cardiac tissue power.

### Clinical implications

During clinical practices of PVI, complications may be reduced by considering an anterior placement of the dispersive electrode, specifically at the hAR position. Following different ablation protocols depending on the tissue thickness seem to be the right direction to improve the efficacy of PVI, despite the marginal 1-year effectiveness improvement when compared to the CLOSE protocol, as shown in the QDOT-by-LAWT trial^30^. Finally, the baseline impedance is not a good indicator for the identification of the tissue power delivery, since comparable values of the baseline impedance may result in much different tissue power dissipation.

### Study limitations

Our patient-specific model does not include yet temperature variations within the tissues. However, while temperature variations would be a more accurate indicator, our previously validated in-silico model mimicking an in-vitro experimental setup shows that dissipated power correlates well with temperature in the tissue^7^. Since the aim of this study is to better understand the impact of the internal body structures in the power dissipation for different DP placements, we expect this aspect to have little impact on our conclusions.

This study did not account for the fiber orientation within the cardiac tissue. Fiber orientation plays a significant role in directing the flow of electrical current, and can influence the power distribution within the tissue. For thin tissues like the left atrial wall, this effect should be less pronounced, and thus we consider using homogenous tissue properties.

Additionally, mechanical deformation occurs due to the contact of the tissue with the catheter, and also due to the heartbeat during the ablation process. These aspects were not considered in this work, under the assumption of small contractions that occur in the left atrium, and also due to the low contact force typically used for ablating thin walls. The incorporation of the electrode-tissue contact deformation is part of our ongoing work.

While we investigated patch placement on the anterior chest (vAL), this location is not clinically feasible due to interference with the 12 lead ECG system. In a purely theoretical scenario, this patch location would have decreased the esophageal power dissipation, while maintaining a tissue power comparable to the one by using hPC location.

Additionally, the study uses a male anatomical model to analyze power dissipation patterns during RFA. Extension to a virtual cohort of patients is required to generalize our results on a wider virtual population.The tissue parameters considered in the presented work are obtained from the ITIS database^27^, the most comprehensive database for human tissue parameters. The study considers average human values, while inherent variability is observed by patient individuality. To quantify the uncertainty in the parameters would require techniques similar to the ones presented in^36^, and is part of our ongoing work.

## Conclusions

The horizontal posterior placement of the patch on the interscapular area of the back provides the largest tissue power dissipation. Placing the dispersive electrode at the lower right side of the anterior thorax reduces the esophageal power dissipation, and provides a small change in the cardiac tissue power dissipation, making it a good candidate location for avoiding esophageal tissue injury. The different patch placement does not significantly alter the localized nature of the treatment. Tissue thickness significantly affects the power dissipation within the cardiac tissue.

## Supplementary Information

Supplementary Information 1.

Supplementary Information 2.

## Data Availability

Data is provided within the manuscript or supplementary information files.
