# Supplementary material for: Impact of the dispersive patch placement on dissipated power in radiofrequency ablation for pulmonary vein isolation via a virtual patient study
Source: Sci Rep. 2025 Feb 27;15:6986. doi: 10.1038/s41598-025-90158-1 (PMC11865282; doi:10.1038/s41598-025-90158-1)
Supplement: Supplementary file 1 — Supplementary Information 1. [file 41598_2025_90158_MOESM1_ESM.pdf]

Supplementary material

Impact of the dispersive patch placement on dissipated power in radiofrequency ablation for pulmonary vein isolation via a virtual patient study

Minha Anees[1], Zoraida Moreno Weidmann[2], David Viladés Medel[2], Jose M. Guerra[2], Luca Gerardo-Giorda[1,3], and Argyrios Petras[1]

[1]Johann Radon Institute for Computational and Applied Mathematics (RICAM), Austrian Academy of Sciences, Linz, Austria
[2]Department of Cardiology, Hospital de la Santa Creu i Sant Pau, IIB Sant Pau, Universitat Autònoma de Barcelona, CIBER CV, Barcelona, Spain
[3]Institute for Mathematical Methods in Medicine and Data-Based Modelling, Johannes Kepler University, Linz, Austria

1 Mathematical model

The electrical potential equation models the electric field distribution in the entire torso geometry, given by

$$-\nabla \cdot \sigma \nabla \Phi = 0,$$

where Φ is the electrical potential and σ is the electrical conductivity of the different subdomains in our geometry.

The RF current is generated by the applied voltage V_0 at the upper boundary of the electrode, similar to[1]. To complete the electric circuit, zero voltage is applied at the dispersive patch ($\Phi = 0$). For the remaining torso boundary and the catheter body, insulation boundary condition ($\partial\Phi/\partial n = 0$) is applied.

To enforce a constant power dissipated within the geometry, the potential equation is augmented with a power constraint,

$$\int_{\Omega} \sigma \nabla \Phi \cdot \nabla \Phi \, dx = P,$$

where P is the power dissipated in the system. The applied voltage V_0 is tuned to match the dissipated power in the system, as detailed in[1].

The power dissipated in a certain area of interest Ω_X is

$$P_X = \int_{\Omega_X} \sigma \nabla \Phi \cdot \nabla \Phi \, dx.$$

In particular, Table 1 shows the different volumes and the corresponding computed power, as referred to in the main manuscript. For the power calculation within radius, the center of the ball is considered as the center of the spherical tip electrode. For tissue power in the neighborhood of the electrode, the radii considered are 2, 3 and 4 times the radius of the electrode (R_{elec} =1.165 mm). On the other hand, for the total power distribution, the radii are chosen as 12.5 mm, 25 mm, 50 mm and 100 mm. The power dissipation observed is confined to the patch's immediate neighborhood, extending up to a radius of 5 mm from its boundary.

Name in manuscript	Tissue power	Tissue power within radius	Total power within radius	Esophageal power	Power around DP
Area of interest Ω_X	Ω_{LA}	$\Omega_{LA} \cap B(\mathbf{C}, r)$	$\Omega \cap B(\mathbf{C}, r)$	Ω_{eso}	$\Omega_{aroundDP}$
Dissipated power P_X	P_{tissue}	P^r_{tissue}	P^r	P_{eso}	$P_{aroudDP}$

Table 1. The power dissipation corresponding to different areas of interest, which include within the left atrial (LA) tissue, the tissue power calculation within a radius, the total power within a radius and the esophageal (eso) power within its wall. The ball $B(\mathbf{C}, r)$ is centered at the center of the electrode $\mathbf{C}$ with a given radius r.

2 Model parameters

The model parameters for the different simulated human tissues were obtained from ITIS database[2], the most comprehensive database for human tissue biophysical parameters, at a frequency of 500 kHz. The electrical conductivity of the lungs was considered to be the average value from inflated and deflated states. The pericardium typically consists of fibrous and serous pericardium, and fat can also infiltrate it. Since no values were found in the literature for its electrical conductivity, we consider the value of fat to be representative of its insulating role.

A composition of 30 % fat tissue and 70 % muscle in the torso is considered, following average values for 60 year old males[3]. This value is applied on the remaining volume after excluding all 34 segmented tissues and organs within the torso.

The electrode electrical conductivity is chosen as in[1]. All model parameters are summarized in Table 2.

Parameters	Conductivity (S/m)
Bone	0.02
Lungs	0.12
Liver	0.14
Stomach	0.55
Esophageal lumen	10^{-5}
Esophageal wall	0.55
Spleen	0.14
Electrode	4.6×10^{6}
Blood (aorta, pulmonary artery, superior vena cava, inferior vena cava, left atrium, right atrium, left ventricle, right ventricle, coronary sinus)	0.74
Cardiac tissue (left atrium, right atrium, left ventricle, right ventricle, left ventricle trabeculae, right ventricle trabeculae, atrial pectinate muscles)	0.28
Pericardium	0.04
Vessel wall (superior vena cava, inferior vena cava, pulmonary veins, pulmonary artery, aorta, coronary sinus)	0.32
Kidney (left, right)	0.22
Pancreas	0.56
Remaining torso	0.24

Table 2. The summary of all electrical conductivities (S/m) incorporated into our torso model.

3 Supplementary results

3.1 Impact of patch location on the power distribution

Figure 1 shows the tissue power dissipated within different radial distances from the electrode for different dispersive patch locations. The patch location appears to have a minimal effect on the power distribution within the cardiac tissue, with variations less than 0.1 %. On the other hand, the power distribution within the tissue has some small variations for all the different electrode locations considered with relative variations from the median value up to 7.5 % for $2R_{elec}$, and 4.5 % for $3R_{elec}$, and $4R_{elec}$, per electrode position. The median, first and third quartiles (Q1 and Q3 respectively), and minimum and maximum values for the relative dissipation (%) of the total power for all radial distances considered are shown in Table 3.

3.2 Tissue power and impedance for high power short duration protocols

Figure 2 shows the tissue power and the baseline impedance for all the different dispersive patch locations and electrode positions considered in the high power ablation protocol. Similar to the standard protocol, no clear correlation is shown between the tissue power and the impedance for a fixed patch location across the different ablation sites. The baseline impedance remains the lowest for hPC patch placement, consistently with the standard ablation case. For a fixed electrode position, an inverse correlation of baseline impedance and tissue power dissipation is observed in both standard and high power protocols, by varying the electrode patch placement.

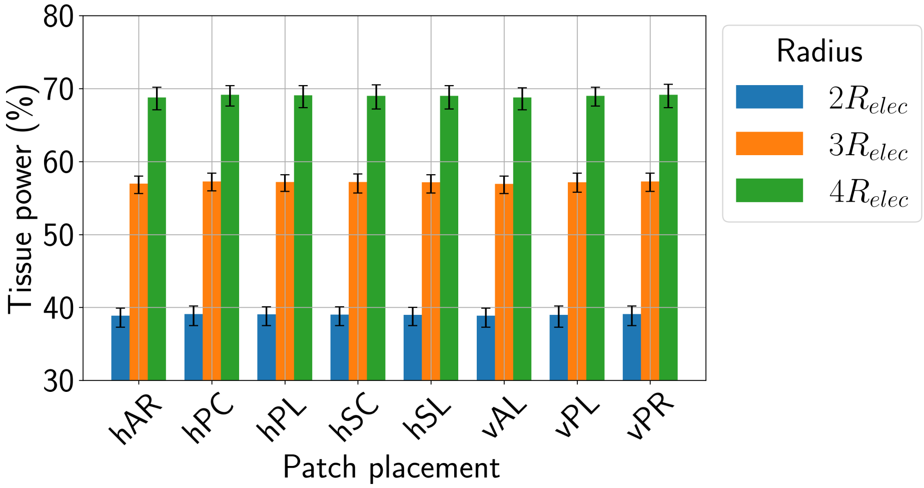

Figure 1. Median tissue power distribution within different radial distances from the electrode across all different considered electrode locations, for different dispersive patch placements. The black lines on the bars denote the corresponding range for the different electrode positions.

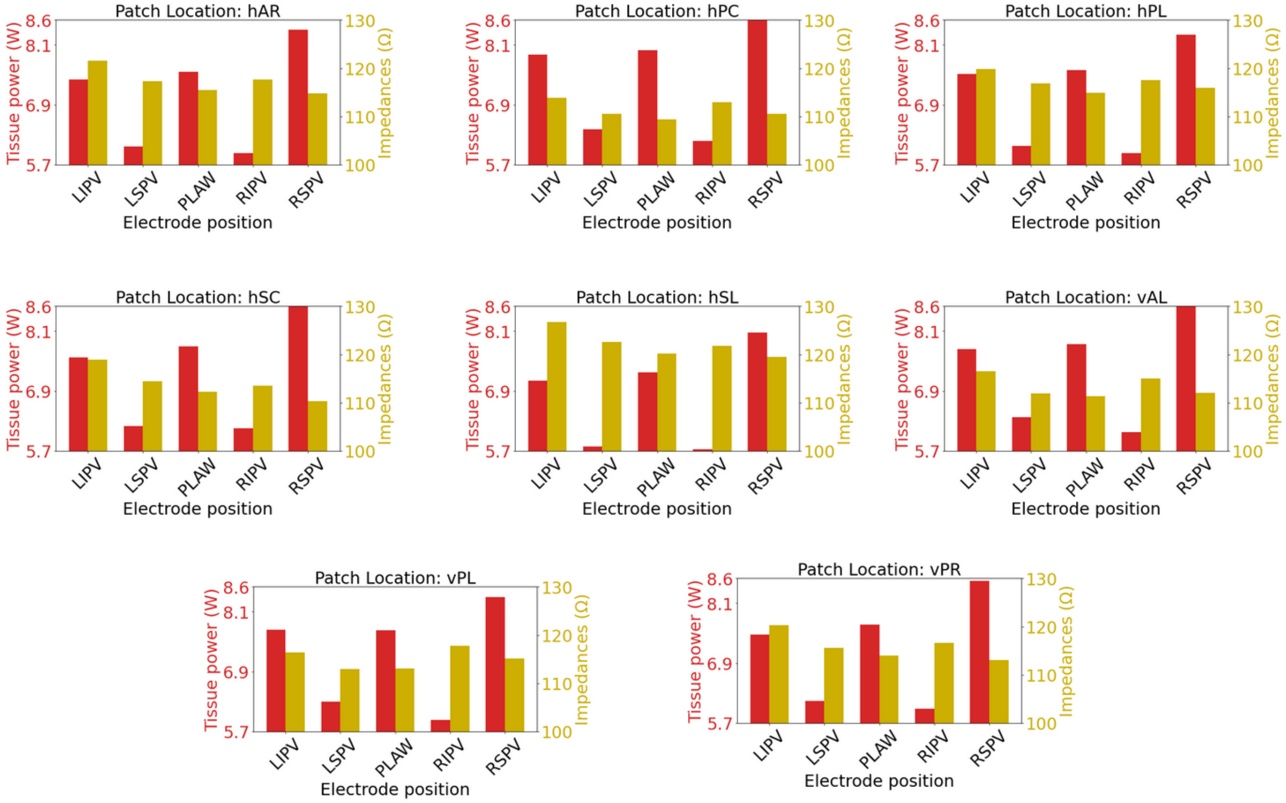

Figure 2. Tissue power and baseline impedance across different electrode positions and patch locations, for the high power ablation protocol. On the left y-axis in red color is tissue power, while on the right y-axis in yellow color is baseline impedance.

Radius	All cases	hAR	hSC	hPL	hPC	hSL	vAL	vPL	vPR
**12.5**	73.7 [72.9 : 75.5]<br>(69 ; 77.2)	72.4 [72.0 : 73.6]<br>(72.0 ; 73.6)	74.8 [74.0 : 76.4]<br>(73.6 ; 76.8)	73.2 [72.8 : 73.2]<br>(72.4 ; 74.0)	76.8 [76.8 : 76.8]<br>(76.8 ; 77.2)	69.6 [69.2 : 70.8]<br>(69.2 ; 71.2)	75.6 [75.2 : 75.6]<br>(75.2 ; 75.6)	74.0 [73.6 : 75.2]<br>(73.6 ; 75.6)	73.6[73.2:74.4]<br>(72.8; 74.8)
**25**	80.6 [79.5 : 82.4]<br>(75.5 ; 84.5)	79.2 [78.8 : 80.0]<br>(78.8 ; 80.4)	81.6 [80.8 : 83.6]<br>(80.8 ; 83.6)	79.6 [79.2 : 80.0]<br>(79.2 ; 80.4)	84.0 [83.6 : 84.0]<br>(83.2 ; 84.4)	76.0 [76.0 : 77.2]<br>(75.6 ; 77.6)	82.4 [82.0 : 82.4]<br>(82.0 ; 82.8)	80.8 [80.4 : 82.4]<br>(80.0 ; 82.8)	80.4 [80.4 : 81.2]<br>(80.0 ; 81.6)
**50**	84.8 [83.5 : 86.5]<br>(79.2 ; 89.2)	83.2 [82.8 : 84.0]<br>(82.8 ; 84.4)	83.6 [83.2 : 84.4]<br>(83.2 ; 84.4)	88 [88.0 : 88.0]<br>(87.2 ; 89.2)	80 [79.6 : 80.8]<br>(79.2 ; 81.6)	86.0 [86.0 : 86.8]<br>(86.0 ; 86.8)	85.2 [83.6 : 87.2]<br>(84.0 ; 86.4)	84.8 [84.4 : 85.6]<br>(84.0 ; 85.6)	84.8 [84.4 : 85.6]<br>(84.0 ; 85.6)
**100**	86 [86.0 : 87.6]<br>(85.6 ; 87.6)	89.6 [88.4 : 92.8]<br>(88.4 ; 94.0)	88.4 [86.8 : 89.2]<br>(86.4 ; 89.2)	93.6 [93.2: 94.4]<br>(91.2 ; 96.0)	83.6 [83.2 : 84.8]<br>(82.4 ; 86.0)	89.2 [83.2 : 84.8]<br>(82.4 ; 86.0)	89.2 [89.2 : 89.6]<br>(89.2 ; 90.0)	90.0 [87.6 : 91.6]<br>(86.8 ; 94.0)	90.0 [88.8 : 90.4]<br>(88.8 ; 90.4)

Table 3. The median, first and third quartiles [Q1 : Q3], and minimum and maximum (min ; max) values of the relative dissipation (%) of the total power for all considered patch locations and electrode positions within different radial distances from the electrode.

References

1. Petras, A., Leoni, M., Guerra, J. M., Jansson, J. & Gerardo-Giorda, L. A computational model of open-irrigated radiofrequency catheter ablation accounting for mechanical properties of the cardiac tissue. *Int. J. for Numer. Methods Biomed. Eng.* **35**, e3232, DOI: 10.1002/cnm.3232 (2019).

2. Hasgall, P. *et al.* IT'IS Database for thermal and electromagnetic parameters of biological tissues, DOI: 10.13099/VIP21000-04-1 (2022).

3. Borrud, L. G. *et al.* Body composition data for individuals 8 years of age and older: Us population, 1999–2004. *Vital health statistics. Ser. 11, Data from national health survey* 1 (2010).
